# Interleukin-22 is elevated in lavage from patients with lung cancer and other pulmonary diseases

**DOI:** 10.1186/s12885-016-2471-2

**Published:** 2016-07-07

**Authors:** Amanda Tufman, Rudolf Maria Huber, Stefanie Völk, Frederic Aigner, Martin Edelmann, Fernando Gamarra, Rosemarie Kiefl, Kathrin Kahnert, Fei Tian, Anne-Laure Boulesteix, Stefan Endres, Sebastian Kobold

**Affiliations:** Division of Respiratory Medicine and Thoracic Oncology, Department of Internal Medicine V, Thoracic Oncology Centre Munich, Ludwig-Maximilians Universität München, Ziemssenstraße 1, 80336 Munich, Germany; Center of Integrated Protein Science Munich (CIPS-M) and Division of Clinical Pharmacology, Department of Internal Medicine IV, Ludwig-Maximilians Universität München, Lindwurmstraße 2a, 80337 Munich, Germany; Department of Medical Informatics, Biometry and Epidemiology, Ludwig-Maximilians Universität München, Munich, Germany; Walter-Straub Institute of Pharmacology and Toxicology, Ludwig-Maximilians Universität München, Munich, Germany; German Center for Lung Research (DZL CPC-M), Munich, Germany

**Keywords:** Bronchoalveolar lavage, Interleukin-22, Biomarker, Lung cancer, Pneumonia

## Abstract

**Background:**

Interleukin-22 (IL-22) is involved in lung diseases such as pneumonia, asthma and lung cancer. Lavage mirrors the local environment, and may provide insights into the presence and role of IL-22 in patients.

**Methods:**

Bronchoscopic lavage (BL) samples (*n* = 195, including bronchoalveolar lavage and bronchial washings) were analysed for IL-22 using an enzyme-linked immunosorbent assay. Clinical characteristics and parameters from lavage and serum were correlated with lavage IL-22 concentrations.

**Results:**

IL-22 was higher in lavage from patients with lung disease than in controls (38.0 vs 15.3 pg/ml, *p* < 0.001). Patients with pneumonia and lung cancer had the highest concentrations (48.9 and 33.0 pg/ml, *p* = 0.009 and *p* < 0.001, respectively). IL-22 concentration did not correlate with systemic inflammation. IL-22 concentrations did not relate to any of the analysed cell types in BL indicating a potential mixed contribution of different cell populations to IL-22 production.

**Conclusions:**

Lavage IL-22 concentrations are high in patients with lung cancer but do not correlate with systemic inflammation, thus suggesting that lavage IL-22 may be related to the underlying malignancy. Our results suggest that lavage may represent a distinct compartment where the role of IL-22 in thoracic malignancies can be studied.

## Background

Interleukin-22 (IL-22) is a cytokine from the interleukin-10 family which acts exclusively on IL22-receptor-1 (IL-22-R1) positive epithelial and endothelial cells [1]. In the lung IL-22 has been shown to be expressed by T cells, natural killer-cells, macrophages, epithelial and potentially also by tumour cells [2]. Its effects can be both immunoregulatory and proinflammatory depending on the stage of disease [3, 4]. IL-22 seems to be protective in the acute phases of lung inflammation or injury such as pneumonia, fungal infection, traumatic lung injury, acute lung injury associated with pancreatitis or the initial phase of allergic airway inflammation [4–8]. In acute inflammation, IL-22 recruits inflammatory cells to clear the infection, probably through the local upregulation of chemokines in the lung, and to rescue lung epithelial cells from cell death [5, 9]. However, if the pathological condition is not cleared and the inflammation becomes chronic, IL-22 seems to sustain inflammation and contribute to the disease phenotype [3, 10]. Recently, we and others have found evidence for IL-22 as a mediator in the interaction between lung cancer cells and the immune environment [11]. In vitro IL-22 promotes tumour growth and chemotherapy resistance of lung cancer cells. Analysis of a large cohort of patients suffering from lung cancer has revealed that IL-22 is frequently expressed in lung cancer tissue, but the clinical significance of these findings has yet to be addressed [12]. In addition, we previously measured IL-22 serum levels in lung cancer patients and matched healthy controls but did not find any difference in spite of strong tissue expression [12]. These observations prompted us to hypothesise that the systemic circulation may not adequately reflect processes in the lung, and that a closer analysis of the pulmonary compartment may help to better understand the role of IL-22 in lung cancer.

In the present study, we analysed lavage specimens from 195 consecutive patients (37 with lung cancer) undergoing clinically indicated bronchoscopy and correlated IL-22 expression with local and systemic cell counts and with serum markers of inflammation.

## Methods

### Study protocol

Patients underwent routine diagnostic or therapeutic flexible bronchoscopy in the Respiratory Medicine and Thoracic Oncology Section of the Internal Medicine Department V, Ludwig Maximilians University of Munich, Germany. Bronchoscopy was carried out under conscious sedation following written informed consent. Bronchoalveolar lavage and bronchial washings, described here together as bronchoscopic lavage (BL), were carried out as indicated, in most cases for diagnostic cytological, pathological or microbiological evaluation. The decision to perform bronchoalveolar lavage vs. washings was at the discretion of the responsible physician. Excess lavage material was used for IL-22 analysis. Patient samples and data were anonymised. Technicians performing the analyses were blinded to all clinical information including patient diagnosis. The study and its protocol were approved by the local ethics board (Ethikkommission der Universität München, decision number EK 376-11).

### Patients and samples

Samples (166 bronchoalveolar lavages and 29 bronchial washings) were collected from 195 patients comprising 83 women and 111 men (one gender not documented), mean age 58.7 years. Patient characteristics are summarized in Table 1. The diagnostic evaluation including bronchoscopy and appropriate imaging, blood work, biopsies and cultures as indicated revealed 47 patients (24 %) with pulmonary infection, of whom three had tuberculosis, two had pneumocystis jirovecii and 42 had other bacterial and viral pneumonias. Thirty-seven patients (19 %) had a diagnosis of lung cancer, with 35 cases of non-small-cell lung cancer and two of small cell lung cancer. Fourteen patients (7 %) had other thoracic malignancies or pulmonary metastases from extrathoracic tumours. Diagnostic work-up revealed 79 patients with other lung diseases (41 %), including three patients with Wegener’s granulomatosis, two patient with chronic graft rejection following lung transplantation, two patients with ARDS, four patients with exogenous allergic alveolitis/hypersensitivity pneumonitis, 20 patients with sarcoidosis and 43 patients with other interstitial lung diseases or fibrosis. Twenty-two patients (11 %) who underwent bronchoscopy due to pulmonary symptoms or suspicion of malignancy on imaging were not diagnosed with a pulmonary disorder following bronchoscopy and clinical work up including appropriate imaging and pulmonary function testing. These patients were used in the analyses as the reference cohort. Because we did not recruit healthy asymptomatic volunteers for bronchoscopy and lavage the reference cohort includes individuals with findings such as benign pulmonary nodules and/or prominent mediastinal lymph nodes, and symptoms such as cough due to vocal cord dysfunction.

**Table 1:** Lavage interleukin-22 concentration in clinically characterized cohorts

Characteristics	Number of patients (% of study cohort)	IL-22 [pg/ml] (median)	Number of samples above DL
Gender			
Female	83 (44 %)	28	65
Male	111 (57 %)	37	82
Age (years)	58.7		
Diagnosis			
Pulmonary Infection	49 (25 %)	49	37
Lung Cancer	37 (19 %)	38	23
Thoracic manisfestation of non-lung cancer	14 (7 %)	33	9
Other lung diseases	79 (41 %)	49	60
Reference cohort	22 (11 %)	15	12

*DL* detection limit of the assay

### Lavage samples and routine analysis

Bronchoalveolar lavage and bronchial washings were collected and analysed according to standard operating procedures at our centre, which are reviewed regularly and are in line with published protocols [13] and indications [14, 15]. In brief, following local anesthesia patients were sedated and intubated nasally with a flexible bronchoscope. For bronchoalveolar lavage the bronchoscope was advanced into wedge position preferentially in the right middle lobe. Normal saline was instilled in 20 ml aliquots to a total volume of 120 to 160 ml and was retrieved using suction. For bronchial washings the bronchoscope was introduced into the area of clinical interest (in most cases the segment thought to be affected by infection or tumour) and normal saline (generally 40 to 80 ml) was instilled and retrieved using suction. A standard morphological and immunologic analysis of BAL cellular components was performed and included total cell count, differential count of macrophages, lymphocytes and neutrophils as well as flow cytometry analysis of the lymphocyte subsets, including BAL CD4/CD8 T-cell ratio. Differential cell count (leukocytes, lymphocytes, neutrophils, macrophages and CD4/CD8 ratio) subgroups were based on accepted cut-off values used for the interpretation of BAL fluid. Bacterial cultures and cytological analyses were performed as clinically indicated at institutes affiliated with the Ludwig-Maximilians Universität in Munich. Analysis of blood samples was performed as part of the routine diagnostic work up at the discretion of the treating physician and in line with national recommendations [16].

### Enzyme-linked immunosorbent assay (ELISA)

ELISA for IL-22 detection was obtained from R&D, Abington, UK. In brief, 50 μl of diluted samples (in triplicates) were loaded and incubated for 2 h at room temperature (RT). Detection antibody was applied for 2 h at RT and streptavidin-bound horseradish peroxidase (HRP) was added for 20 min at RT. Absorption was measured at 450 nm using a Mithras reader (Berthold Technologies, Bad Wildbad, Germany). The detection limit of the ELISA was 15 pg/ml.

### Statistics and data analysis

For the IL-22 levels, mean values of three independent experiments each performed in triplicates were calculated and used for subsequent analysis. The differences in IL-22 levels between two independent groups were assessed using the two-part Wilcoxon test [17], in which the values below the detection threshold 15 were set to 0. Similarly, correlations between IL-22 levels and other continuous variables (CRP, leucocytes, lymphocytes, neutrophiles, macrophages, eosinophiles, CD4/CD8) were assessed using Spearman’s rank-based correlation test with values of Interleukin-22 below 15 set to 0. *P*-values < 0.05 were considered as significant. Statistical analyses were performed using R 3.0.2.

Samples from patients with lung cancer suffering from chronic obstructive pulmonary disease (COPD) or lung infection were excluded from the comparative analysis with the control cohort and correlation with clinical parameters to avoid bias in the IL-22 concentrations due to causes other than lung cancer (*n* = 16, 45 %).

## Results

### IL-22 is elevated in bronchoscopic lavage from patients with lung cancer

**Fig. 1:**
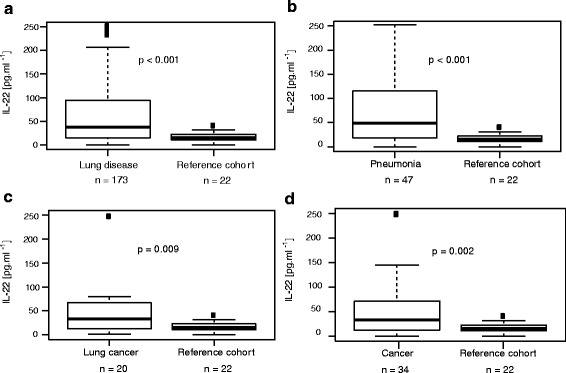
IL-22 concentrations in lavage are higher in patients with lung cancer. **a** Comparison between BL IL-22 concentrations found in *n* = 173 bronchoscopic lavage (BL) samples from patients with lung disease and controls (*n* = 22). **b** Comparison between BL IL-22 concentrations for samples from patients with pneumonia (*n* = 47) and controls (*n* = 22). **c** Comparison between BL IL-22 concentrations of patients with lung cancer (*n* = 20) and controls (*n* = 22). Samples from lung cancer patients with a known coexisting inflammatory lung pathology such as COPD or lung infection were excluded from this analysis to avoid confounding due to additional inflammation. **d** Comparison between BL IL-22 concentrations for samples from patients with lung cancer and thoracic manifestations of other malignancies, summed up as “cancer” (*n* = 34) and controls (*n* = 22). *P*-values were calculated using the two-part Wilcoxon test after setting all values <15 to 0

Patients with confirmed lung disease (*n* = 173) had significantly higher IL-22 levels in bronchoscopic lavage (BL) than the reference cohort (38 vs 15 pg/ml, *p* < 0.001, Fig. 1a). The detailed characteristics of the whole cohort are found in Table 1. We could not find any correlation between IL-22 in BL and gender. IL-22 concentrations were higher in patients with pneumonia than in controls (49 vs 15 pg/ml, *p* < 0.001 Fig. 1b). As IL-22 is known to be elevated by acute or chronic inflammation, as seen in pneumonia, we excluded patients with known inflammatory lung diseases from the group of lung cancer patients for further analysis. Patients with lung cancer had high levels of IL-22 compared to the reference cohort (33 vs 15 pg/ml, *p* = 0.009 Fig. 1c). We then extended the cohort to patients with thoracic manifestations of other malignancies, and found that IL-22 concentrations were still elevated compared to controls (33 vs 15 pg/ml, *p* = 0.002, Fig. 1d).

### IL-22 does not correlate with systemic inflammation

To investigate whether IL-22 is a marker of lung disease and especially of lung cancer or rather a reflection of systemic inflammation, we next analysed the relationship between IL-22 and systemic parameters of inflammation. In patients with lung cancer, we were unable to find a relationship between IL-22 levels, systemic leukocyte, lymphocyte or neutrophil counts and CRP (Table 2, c, e, g, *p* = 0.19, 0.33, 0.28 and 0.35, respectively). We also investigated potential differences in IL-22 biology in the largest disease subgroup (pneumonia) by investigating possible correlations with IL-22 in BL from these patients. We did not find any evidence of a link between IL-22 and systemic inflammation in pneumonia. No correlation was found between IL-22 in BL of patients with pneumonia and systemic leukocyte, lymphocyte or neutrophil counts and CRP (Table 2, *p* = 0.16, 0.21, 0.77 and 0.3, respectively). These results support the notion that IL-22 in BL of lung cancer does not reflect systemic inflammation. However, the power of these correlation analyses was moderate to low due to the limited size of the groups, in particular for parameters with large proportions of missing values.

**Table 2:** Correlation of IL-22 concentrations in lavage with systemic inflammation parameters in samples from patients with pneumonia or lung cancer. Leucocyte subpopulations were not measured in all patients. Correlation was analyzed using Spearman’s rank-based correlation test after setting all IL-22 values <15 to 0

Correlation of IL-22 in lavage with markers of systemic inflammation	Subgroup size (n)	*p*-value for correlation
Patients with Lung Cancer		
Correlation of IL-22 in BL with CRP	16	0.19
Correlation of IL-22 in BL with Leucocytes	16	0.33
Correlation of IL-22 in BL with Lymphocytes	6	0.28
Correlation of IL-22 in BL with Neutrophils	6	0.35
Patients with Pneumonia		
Correlation of IL-22 in BL with CRP	30	0.16
Correlation of IL-22 in BL with Leucocytes	30	0.21
Correlation of IL-22 in BL with Lymphocytes	18	0.77
Correlation of IL-22 in BL with Neutrophils	19	0.30

### IL-22 is not associated with a particular cell type in lavage from patients with lung cancer or pneumonia

To identify potential sources of IL-22 within the lung compartment resulting in elevated IL-22 levels, we correlated IL-22 levels with the measured cellular populations found in lavage. In lung cancer patients, we found no correlation between IL-22 total percentages of lymphocytes, macrophages, neutrophils, eosinophils or the CD4 to CD8 T cell ratio (Table 3; *p* = 0.66, 0.59, 0.53, 0.95, 0.051, respectively). In lavage from patients with pneumonia, IL-22 concentrations were unrelated to total percentages of lymphocytes, macrophages, neutrophils, eosinophils and to the CD4 to CD8 T cell ratio (Table 3, *p* = 0.5, 0.98, 0.86, 0.98, 0.65, respectively). These results may indicate that IL-22 does not originate from a single cell population, should, however, be interpreted with caution due to the limited size of the subgroups.

**Table 3:** Correlation of IL-22 concentrations in lavage with distinct cell populations in the lavage samples of patients with pneumonia or lung cancer. Not all lavage cell populations were measured in all patients. Correlation was analyzed using Spearman’s rank-based correlation test after setting all IL-22 values <15 to 0

Correlation of IL-22 in lavage with cell populations in lavage	Subgroup size (n)	*p*-value for correlation
Patients with Lung Cancer		
Correlation of IL-22 in BL with BL Lymphocytes	9	0.66
Correlation of IL-22 in BL with BL Macrophages	9	0.59
Correlation of IL-22 in BL with BL Neutrophils	9	0.53
Correlation of IL-22 in BL with BL Eosinophils	9	0.95
Correlation of IL-22 in BL with BL CD4/CD8 ratio	4	0.051
Patients with Pneumonia		
Correlation of IL-22 in BL with BL Lymphocytes	32	0.5
Correlation of IL-22 in BL with BL Macrophages	32	0.98
Correlation of IL-22 in BL with BL Neutrophils	26	0.86
Correlation of IL-22 in BL with Eosinophils	26	0.98
Correlation of IL-22 in BL with BL CD4/CD8 ratio	34	0.65

## Discussion

The present study demonstrates that IL-22 concentration in pulmonary lavage samples can be quantified using ELISA and that the levels of IL-22 in lavage vary between different disease entities. Patients with bacterial pneumonia, lung cancer or pulmonary manifestations of other tumours appear to have higher levels of IL-22 in lavage samples compared with non-lung disease controls. In patients with lung cancer, IL-22 levels in lavage did not correlate with systemic signs of inflammation. We found that within the lung, IL-22 may originate from different cell populations.

The finding that patients with NSCLC show higher levels of IL-22 in pulmonary lavage specimens is in line with the results of a previous study reporting that lung cancer cells may produce IL-22 [11]: Zhang and colleagues found high expression of IL-22 in primary tumour tissue, serum, and malignant pleural effusion of NSCLC patients, as well as expression of the IL-22 receptor (IL-22-R1) on lung cancer cell lines. We recently studied the expression of IL-22 in tissue microarray samples of a large cohort of lung cancer patients and found IL-22 expression mostly in patients with large cell NSCLC and those with small cell lung cancer 22 [12]. The lung cancer patient cohort in the present study is, however, too small to confirm these histological subgroup results. In the present study, we did not detect IL-22 in BAL samples from some of the lung cancer patients studied. While technical reasons may be put forward to explain these results, levels of IL-22 may vary significantly between tumor patients. Low levels of IL-22 may be of prognostic relevance, as IL-22 is thought to promote a more aggressive lung cancer phenotype [12].

To the best of our knowledge, the present study is the first to investigate IL-22 concentrations in pulmonary lavage from cancer patients and show that IL-22 is elevated in lavage samples from lung cancer patients. It thus strengthens the hypothesis that IL-22 has a role in lung cancer. The elevated IL-22 levels found in patients with pulmonary manifestations of extrathoracic tumours support the hypothesis that IL-22 may be a mediator in cancer development and progression [18].

In addition to the data we have presented, IL-22 has been investigated in other non-malignant lung diseases. A recent study in pulmonary lavage samples from patients with bronchial asthma revealed that IL-22 concentrations are elevated, further supporting that IL-22 is a disease-associated cytokine detectable in lavage and associated with lung inflammation, as seen in our study [19]. Studies on the host response towards bacterial or fungal pneumonia have revealed that IL-22 contributes both to the acute phase, where it supports clearance, and to the chronic phase, where is prolongs inflammation. In chronic infections such as tuberculosis, IL-22 seems to play a disease promoting role [20]. Our study detected higher levels of IL-22 in lavage samples from patients with pneumonia compared to controls, supporting the suggestion that IL-22 plays a role in the pulmonary response to infection. This is in line with previously published data which identified IL-22 producing cells in BAL from patients with pneumonia [21]. Recently, IL-22 has been detected in lavage samples from patients with community-acquired pneumonia and correlated with serum levels of IL-22, corroborating our finding that IL-22 is associated with pulmonary inflammation [21].

In the lung, IL-22 is thought to be mainly produced by lymphoid cells, among others by CD4-positive lymphocytes [5, 22, 23]. In previous studies analysing lavage samples in patients with tuberculosis, T helper cells were identified as the major source of IL-22 production in BAL [22]. However, in our current study, we did not find any association between IL-22 production, and lymphocytes found in lavage from patients with pneumonia and the number of patients with lung cancer was too low to draw firm conclusions from the analysis with CD4 to CD8 T cell ratio. While T cells as source of IL-22 in lung cancer have not been proposed prior to our study, our data are supported by evidence from other tumor types such as colon, gastric or hepatic carcinomas where CD4 T cells are thought to be the main sources of IL-22 [24–26]. In contrast, in lung cancer, the source of IL-22 remains uncertain. One study has proposed that IL-22 is expressed by the tumor cells themselves [11]; however, an earlier study from our group was not able to confirm these results, supporting the notion that IL-22 is expressed in the environment but not in the tumor cells themselves [12]. This idea is promoted by a recent study analysing IL-22 producing cells in malignant pleural effusions which identified CD4 T cells as major source [27]. Our study may point towards a role for different cell populations rather than an individual one as source of IL-22 in the lung, as we found no clear correlation between IL-22 and the analysed cell populations. However, the current study was not powered to prove a lack of association between cell populations and IL-22 in the lung.

A noteworthy finding of the present study is the dissociation between local IL-22 concentrations and systemic parameters of inflammation such as CRP and leukocyte counts in patients with lung cancer. This suggests that IL-22 in the lavage of lung cancer patients reflects local processes in the lung rather than systemic inflammation. The concept of the airway as a distinct biological compartment with cytokine levels differing from those in the systemic circulation is supported by other studies: Hollander et al. [28] found that the concentrations of IL-8 and of other markers of inflammation were significantly higher in BAL samples compared to serum samples in patients with bronchial asthma and COPD.

Lavage is a clinically useful tool in the diagnostic evaluation of many patients with pulmonary disease and malignancy; however, clinicians must consider the limitations of this technique when interpreting results. The sensitivity of lavage cytology for lung cancer is reported to be 48 %, lower than that of brushings or endobronchial biopsy [29]. Biomarkers in lavage have the potential to improve the sensitivity of this minimally invasive method in lung cancer diagnosis. Techniques to increase the concentration of IL-22 in lavage samples, such as the use of small volume lavage or lavage catheters placed near the tumor, may further increase the diagnostic sensitivity.

## Conclusion

IL-22 can be measured in lavage samples, and correlates with the presence of lung disease. Lavage IL-22 concentrations are highest in patients with pneumonia and lung cancer. IL-22 in lavage does not significantly correlate with systemic inflammation. IL-22 concentrations were not significantly associated with a particular cell population found in the lavage, indicating that IL-22 production may arise from different sources. Our results suggest that IL-22 in pulmonary lavage may serve as a marker for lung cancer, and, perhaps, for pulmonary metastases of other tumours. Markers expressed in the pulmonary compartment can be sampled using bronchoscopic lavage, and may mirror local disease states.
